# Biochemical markers as diagnostic/prognostic indicators for ischemic disease

**DOI:** 10.4314/ahs.v18i2.13

**Published:** 2018-06

**Authors:** MA Alkireidmi, FA Al-Abbasi, MG Mehanna, Said S Moselhy

**Affiliations:** 1 Department of Biochemistry, Faculty of science, King Abdulaziz University, Jeddah, Saudi Arabia; 2 Department of Biochemistry, Faculty of science, Ain Shams University, Cairo, Egypt; 3 King Saud bin Abdul Aziz university for health sciences, college of science and health professions, Basic science department. Jeddah, Saudi Arabia

**Keywords:** Serum enolase, ischemia, hypertension, diagnosis, prognostic

## Abstract

**Objective:**

The use of a biomarker was extremely useful in clinical emergencies such as stroke to aid in triage and early management of cases. The diagnostic accuracy of laboratory biomarkers is run to approve the identification of easy, cheap and fast tests associated with cerebral ischemia and intracranial hemorrhage. The present study was designed to screen serum enolase activity, activities of CK-BB, LDH and lipid profile in patients with ischemic or related diseases as good diagnostic/ prognostic indicator for ischemic diseases.

**Methods:**

Sixty male subjects in the age range of (45 ±2years) were divided into four groups each with 15 participants: Group (I) normal . Group (II) patients recently diagnosed as ischemic disease; Group (III) hypertensive patients and Group (IV); diabetic patients enolase activity (p<0.001) and CK-BB (p<0.01) in ischemic and hypertensive patients compared with control and diabetic groups. LDH level was significantly elevated in ischemic, hypertensive and diabetic patients compared with controls (p<0.001). The cut -off value for serum enolase was 62.5 nmol/l showing 90% sensitivity and 93% specificity for differentiation of ischemic disease. Positive correlations were observed between serum enolase (r = 0.56), and CK-BB (r = 0.53).

**Conclusion:**

Serum enolase can be considered as a more sensitive and specific marker and used as a sensitive diagnostic or prognostic marker for ischemic related diseases.

## Introduction

Transient ischemic attacks (TIA) are episodes in which a person has signs or symptoms of a stroke (e.g. numbness; inability to speak) that last for a short time^1^, but without any sign of stroke on brain scans such as MRI or CT. Symptoms of a TIA usually last between a few minutes and a few hours. A person may have one or many TIAs. People recover completely from the symptoms of a TIA. TIA is a warning sign that a person is at high risk for a stroke; immediate treatment can decrease or eliminate this risk^2^.

Enolase is a glycolytic enzyme that catalyzes the conversion of 2-phosphoglycerate to phosphoenolpyruvate^3^. Enolase exists in the form of several tissue-specific isoenzymes, consisting of homo or heterodimers of 3 different monomer-isoforms (alpha, beta, and gamma)^4^. Neuron specific enolase (NSE) is a 78 kD gamma-homodimer and represents the dominant enolase-isoenzyme found in neuronal and neuroendocrine tissues^5^. Its levels in other tissues, except erythrocytes, are negligible. The biological half-life of NSE in body fluids is approximately 24 hours. Due to this organ-specificity, concentrations of NSE in serum or, more commonly, cerebrospinal fluid (CSF), are often elevated in diseases which result in relative rapid (hours/days to weeks, rather than months to years) neuronal destruction. Measurement of NSE in serum of CSF can therefore assist in the differential diagnosis of a variety of neuron-destructive and neuro-degenerative disorders. The most common application is in the differential diagnosis of dementias, where elevated CSF concentrations support the diagnosis of rapidly progressive dementias. NSE might also have utility as a prognostic marker in neuronal injury^6^.

The evaluation of Neuron specific enolase level in serum and cerebrospinal fluid following cerebral ischemia provides a reliable bio-indicator of the degree of brain cell damage, and may allow for early prediction of outcome^7^. In case of stroke, the first enolase peak within 7–18 h is found following admission and may reflect the initial damage to neuronal tissue, while a second elevation between days 1–3, may be related to edema and an increase in intracranial pressure^8^–^10^. For that, enolase can be used as a reflection of neuronal damage. In this study we aimed to find the correlation between serum enolase, LDH, CK-BB levels in patients with ischemia, hypertension and diabetes. This is promising as a good indicator to avoid ischemic disease and tissue damage.

## Subjects and methods

This study was approved by the ethics committee of the King Abdul-Aziz University Hospital. A written informed consent was obtained from all participants prior to enrollment into the study. Sixty adult males volunteers were included in the present study, age ranging between 40–55 years. The subjects were divided into four groups each with 15: Group (I) normal subjects not suffering from any systemic diseases; Group (II) including patients recently diagnosed as ischemic disease; Group (III) including hypertensive patients (BP ≥ 140/90) and Group (IV) including patients with type II diabetes, fasting HAB1C>10%.

Inclusion criteria were: chronic fatigue, diabetes, heart condition, hypertensive and stroke. Exclusion criteria were: Hormone replacement therapy, multiples sclerosis, oral inflammation, advanced periodontitis and severe gingivitis. Five ml blood samples were collected from all subjects following an overnight 12 hours fasting. Immediately following collection, samples were centrifuged at 8000 rpm at −4°C for separation of serum.

### Biochemical assays

Lipid profile, total cholesterol, LDL-c, HDL-c, enolase activity, Lactate LDH and CK-BB were measured in serum samples using available kits from BIOLINE (England).

### Statistical analysis

Statistical analysis was performed using the SPSS package [version 20]. Results were expressed as mean ± SD. A p value ≤0.05 was considered significant. Using analysis of variance (ANOVA), there was a high significance between the groups when comparing sample.

## Results

**Fig. (1):**
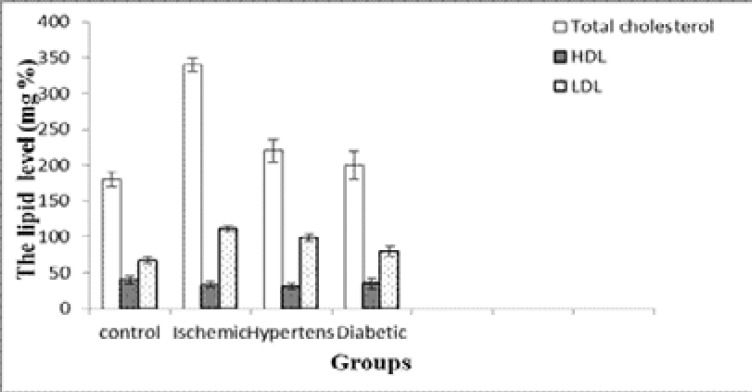
Serum TC, LDL-c and HDL-c levels in the different groups (Mean ± SD).

Results obtained in figure 1 show that, there was a significant elevation in the level of serum total cholesterol in ischemic, hypertensive and diabetic groups compared with the control group (p <0.001, <0.01 and <0.05) respectively. The elevation in hypertension was more than in diabetes. Serum HDL-c level was significantly lower in ischemic, hypertension and diabetic groups compared with the normal group (p <0.001, <0.01 and <0.05) respectively. Serum LDL-c level was significantly increased in ischemic, hypertension and diabetic compared with the control group (p <0.001, <0.01 and <0.05) respectively. While there was no significant difference in the level of LDL-c between the ischemic, hypertension and the diabetic groups.

**Fig. (2):**
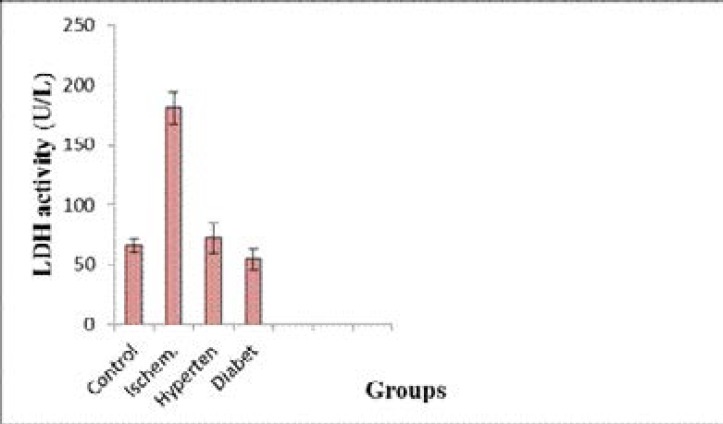
Serum LDH activity in the different groups (Mean ± SD).

**Fig. (3):**
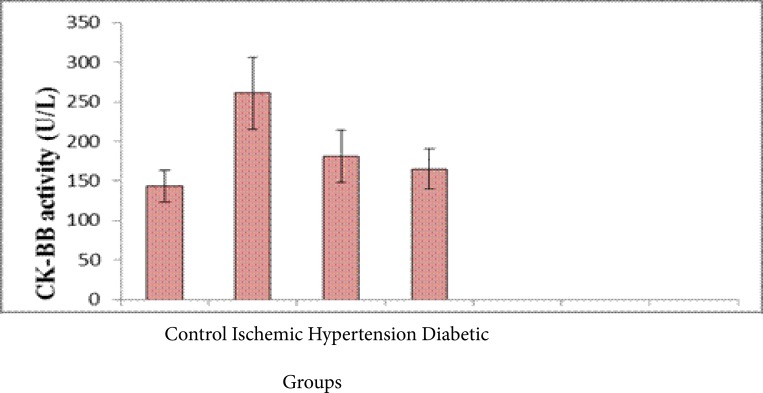
Serum CK activity in the different male rat groups (Mean ± SD).

Figure 2 shows that, Serum lactate dehydrogenase activity was significantly increased (p <0.001)in both ischemic, hypertension and diabetic groups compared with the control group while serum creatine kinase (CK-BB) showed a higher values in both hypertension and diabetic groups compared to control and stroke groups (p <0.001 and <0.001). However it was highest in the hypertension, (p <0.001) and slightly increased in the diabetic group compared with the control group (p <0.05) Figure 3.

**Fig. (4):**
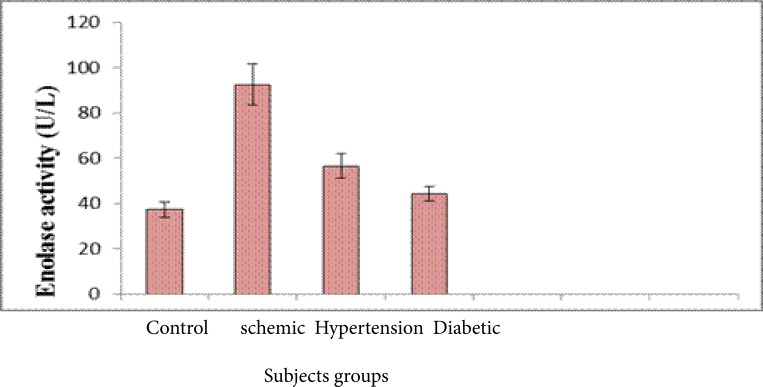
Serum enolase activity in the different groups (Mean ± SD).

Figure 4 reveals that, Serum enolase activity was significantly highest in the hypertension, ischemic (p <0.001, <0.001) and slightly increased in the diabetic group compared with the control group (p <0.05), however the ischemic elevation was similar to the hypertension group and in diabetics, it was lower. Receiver operating curve (ROC) analysis for serum enolase in different groups showing an AUC of 0.92 with a cutoff value of 10% (sensitivity, 95.5%; specificity, 97%). It was increased about 8 folds in ischemic and hypertension group as compared with the control and diabetic groups. However, it was elevated about 5 folds in diabetic group compared to the control group. The cut-off value in 92% ischemic patients was 80 U/L, 80% of hypertensive patients. Positive correlations were observed between serum enolase and CK-BB (r=0.53) while were not correlated with other laboratory markers such as plasma LDH and lipid profile.

## Discussion

The blood-brain barrier is compromised in patients with stroke. Neuron-specific markers, such as neuron specific enolase (NSE) in the circulation may allow the pathophysiology and prognosis of patients with cerebrovascular diseases to be evaluated^11^–^16^. The present study was designed to measure NSE in serum of patients with ischemic stroke and patients with related diseases as a diagnostic tool for early prediction of ischemic stroke. The diagnostic accuracy of laboratory biomarkers is run to approve the identification of easy, cheap and fast test associated with cerebral ischemia and intracranial hemorrhage.

In the present study, results revealed that there was a significant increase in the level of serum total cholesterol in ischemic, hypertension and diabetic groups compared with the control group. However, a significant increase of serum LDL-c in the ischemic, hypertension and diabetic groups compared with the normal group. Weak positive correlations between saliva and serum cholesterol HDL-c was found. Salivary and serum HDL-c concentration recorded in this study may reflect the protective function of HDL-c, since it was markedly reduced in patients with stroke as well as patients with stroke- related diseases. This is in accordance with a study that stated a significant elevation of LDL and a significant decrease in HDL in hypertensive and diabetic patients as compared to controls^17^–^20^. There are several possible mechanisms by which serum lipids can reach saliva. Within the salivary glands, transfer mechanisms include intracellular and extracellular routes^21^,^22^. Results obtained indicated that a significant elevation in the levels of total LDH and CK-BB in serum of ischemic, hypertensive and diabetes versus controls. It was concluded that, the elevation of serum enolase level may be implicated as a predisposition for incidence of hypertension and diabetes and increases the risk of stroke^23^–^25^. The measurement of NSE concentrations in serum and cerebrospinal fluid (CSF) following cerebral ischemia and traumatic head injury provides a reliable laboratory indicator of the degree of brain cell damage, may allow for early prediction of outcome and may reflect the damage to neuronal tissue. Therefore, NSE concentrations can provide early information about neuronal damage.

The present study was designed to measure NSE in serum as early diagnosed in patients with stroke and patients with stroke-related diseases.

However, the concentrations of serum NSE in hypertensive and diabetic patients were significantly increased. This indicated that diabetic patients showed some sort of neuronal damage and/or blood-brain barrier disruption. This finding was in agreement with^26^ who demonstrated a significant increase in antibodies against NSE in both type 1 and type 2 diabetic subjects compared to healthy controls. In hypertensive patients and patients with ischemic heart disease, the mean serum NSE ranked an intermediate value between ischemic stroke and healthy controls.

The presence of NSE in saliva of patients with stroke and ‘at risk’ patients may be explained by the fact that the integrity of the blood-brain barrier is disrupted to various degrees in these patients, and leakage of this enzyme outside the CNS can be detected in salivary secretion. Recently, the determination of SE has been described as a sensitive and easy-to-perform marker for the diagnosis of ischemic for early prediction of occurrence

Positive correlations were observed between serum enolase (r = 0.56), and CK-BB (r = 0.53).

## Conclusion

Serum enolase can be considered as a more sensitive and specific marker and used as a sensitive diagnostic or prognostic marker for ischemic related diseases.

## Figures and Tables

**Fig 5:**
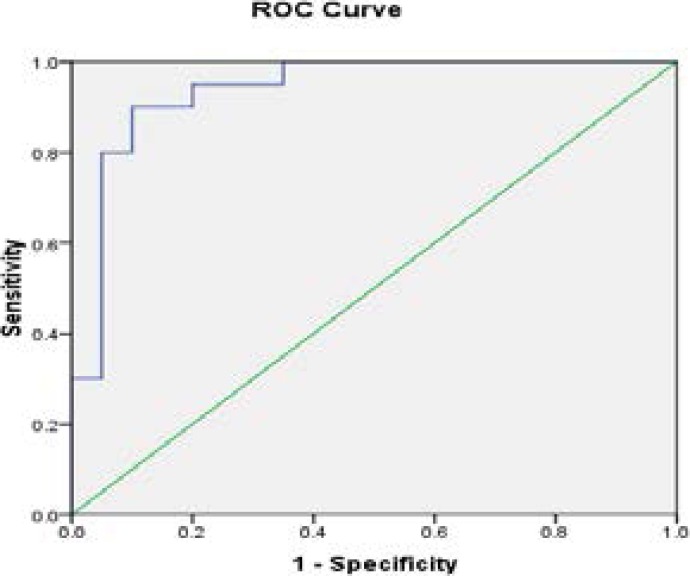
The ROC curve of Ischemic versus control

**Fig 6:**
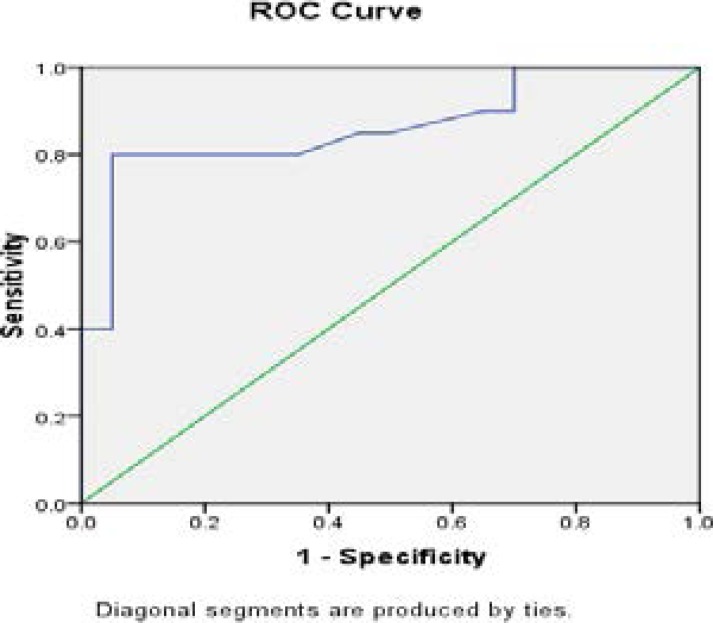
The ROC curve of Hypertension versus Control
